# Longitudinal genomic surveillance of carriage and transmission of *Clostridioides difficile* in an intensive care unit

**DOI:** 10.1038/s41591-023-02549-4

**Published:** 2023-09-18

**Authors:** Arianna Miles-Jay, Evan S. Snitkin, Michael Y. Lin, Teppei Shimasaki, Michael Schoeny, Christine Fukuda, Thelma Dangana, Nicholas Moore, Sarah E. Sansom, Rachel D. Yelin, Pamela Bell, Krishna Rao, Micah Keidan, Alexandra Standke, Christine Bassis, Mary K. Hayden, Vincent B. Young

**Affiliations:** 1 Department of Microbiology and Immunology, University of Michigan Medical School, Ann Arbor, MI USA; 2 Division of Infectious Diseases, Department of Internal Medicine, University of Michigan, Ann Arbor, MI USA; 3 Division of Infectious Diseases, Department of Medicine, Rush University Medical Center, Chicago, IL USA

**Keywords:** Epidemiology, Policy and public health in microbiology, Pathogens

## Abstract

Despite enhanced infection prevention efforts, *Clostridioides difficile* remains the leading cause of healthcare-associated infections in the United States. Current prevention strategies are limited by their failure to account for patients who carry *C. difficile* asymptomatically, who may act as hidden reservoirs transmitting infections to other patients. To improve the understanding of asymptomatic carriers’ contribution to *C. difficile* spread, we conducted admission and daily longitudinal culture-based screening for *C. difficile* in a US-based intensive care unit over nine months and performed whole-genome sequencing on all recovered isolates. Despite a high burden of carriage, with 9.3% of admissions having toxigenic *C. difficile* detected in at least one sample, only 1% of patients culturing negative on admission to the unit acquired *C. difficile* via cross-transmission. While patients who carried toxigenic *C. difficile* on admission posed minimal risk to others, they themselves had a 24-times greater risk for developing a healthcare-onset *C. difficile* infection than noncarriers. Together, these findings suggest that current infection prevention practices can be effective in preventing nosocomial cross-transmission of *C. difficile*, and that decreasing *C. difficile* infections in hospitals further will require interventions targeting the transition from asymptomatic carriage to infection.

## Abstract (web summary)

A low rate of *Clostridioides difficile* transmission within an intensive care unit supports the effectiveness of prevention practices and highlights the greater risk to carriers of toxigenic *C. difficile*.

## Main

*Clostridioides difficile* is the most common cause of healthcare-associated infectious diarrhea and is responsible for substantial morbidity, mortality and healthcare costs in the United States each year^1–3^. Recent molecular epidemiological studies indicated that a minority of *C. difficile* infections (CDIs) can be linked to other symptomatic CDI cases in the same hospital, suggesting that there are uncharacterized healthcare or community-based reservoirs of *C. difficile*^4,5^. To make further gains in reducing rates of *C. difficile* infection, it is necessary to better understand the sources of *C. difficile* beyond cases with CDI within the hospital.

Asymptomatic carriers of *C. difficile*, defined as persons who carry the *C. difficile* organism without clinical symptoms indicative of *C. difficile* infection, could be underappreciated reservoirs of *C. difficile* within healthcare settings^6,7^. Asymptomatic carriers are more common in the hospital than symptomatic patients: as many as 29% of high-acuity patients in acute care settings have been shown to carry *C. difficile* asymptomatically^8^. The risk of transmission from unidentified, asymptomatic carriers may also be higher than from symptomatic patients because carriers can shed spores into the environment, yet they are usually not under the same contact precautions and their rooms may not undergo the same environmental cleaning procedures as patients with CDI^9,10^. Additionally, recent data indicated that carriers of *C. difficile* are at a higher risk of developing CDI than noncarriers^11^. However, the risk that asymptomatic carriers of *C. difficile* pose, both to other patients and to themselves, is incompletely characterized due to a lack of available data collected via detailed longitudinal sampling and high-resolution typing. Thus, the utility of screening patients for *C. difficile* on admission—both to prevent transmission and infection—remains under debate^10^ and professional medical societies have not issued recommendations regarding screening patients for asymptomatic carriage of *C. difficile*^12^.

In this study, we sought to characterize the burden, dynamics and risk factors associated with *C. difficile* carriage in a US-based medical intensive care unit (ICU). To accomplish this, we used admission and prospective daily culture-based screening for *C. difficile* over nine months and applied whole-genome sequencing (WGS) to all identified isolates. We assessed epidemiological evidence of *C. difficile* importation and acquisition, examined genomic evidence of *C. difficile* transmission from *C. difficile* carriers and described the association between *C. difficile* importation and CDI during hospitalization.

## Results

### Cohort description

**Fig. 1:**
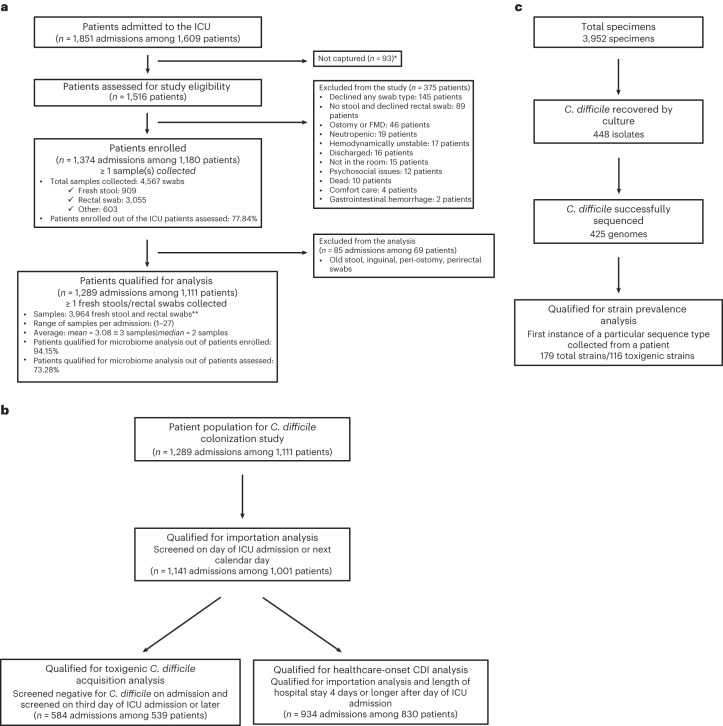
Flow diagram of study enrollment and inclusion criteria. **a**, Overall study. FMD, fibromuscular dysplasia. **b**, Admission-level analyses. **c**, Strain-level analyses.

In this longitudinal, observational, single-center study, we collected 3,952 rectal swab and stool samples from 1,289 unique ICU admissions and 1,111 unique patients; 448 *C. difficile* isolates were recovered via enrichment culture for toxigenic and non-toxigenic strains and 425 of those were successfully whole-genome sequenced on an Illumina NovaSeq instrument (Fig. 1 and Supplementary Information). A median of two samples were collected per ICU admission (interquartile range (IQR) = 1–3 samples), and the median length of stay per admission was 3 days (IQR = 2–6 days). The basic demographics and clinical characteristics of the entire screened cohort, shown in Table 1, reflected older patients (mean age = 62.7 years), with Black or African American as the most listed ethnicity (43.8% of admissions), a high burden of underlying medical conditions (median Charlson Comorbidity Index score of 4), high exposure to antibiotics (71.8% during ICU admission) and most admissions through an emergency department (ED).

**Table 1:** Selected patient characteristics from all ICU admissions included in this study

Characteristics	*n* = 1,289
Age, years, median (IQR)	62.7 (50.0–72.7)
Male sex, *n* (%)	628 (48)
Ethnicity, *n* (%)	
White	486 (37.7)
Black or African American	565 (43.8)
Asian	27 (2.1)
Native Hawaiian or Other Pacific Islander	1 (0.1)
American Indian or Alaska Native	4 (0.3)
Other	187 (14.5)
Unknown	19 (1.5)
Ethnic group, *n* (%)	
Not Hispanic or Latino	1023 (79.4)
Hispanic or Latino	258 (20)
Unknown	8 (0.6)
Median ICU length of stay, days	3.0 (2.6)
Admission source, *n* (%)	
Same or different hospital ED	724 (56.2)
Different ward in same hospital	273 (21.2)
Different hospital, LTACH, NH, SNF, ICF	207 (16.1)
Other^**a**^	85 (6.6)
Charlson Comorbidity Index	4 (2.6)
Antimicrobial exposure during ICU admission, *n* (%)	926 (71.8)

^a^Includes admission from clinic/physician’s office, non-healthcare facility, or home. ICF, intermediate care facility; LTACH, long-term acute care hospital; NH, nursing home; SNF, skilled nursing facility.

### Detected *C. difficile* strains showed substantial diversity

**Fig. 2:**
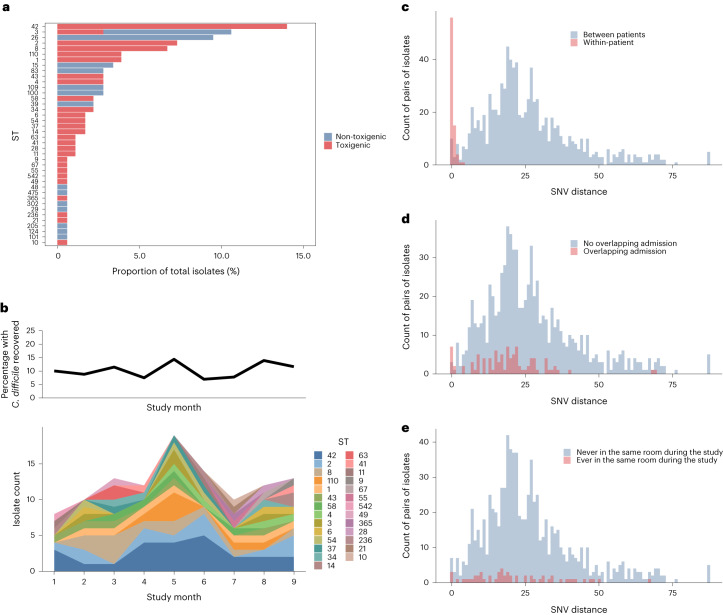
*C. difficile* strain diversity in the overall patient population and between epidemiologically linked carriers. **a**,**b**, ST diversity shown cumulatively during the study period (**a**), colored according toxigenic (red) and non-toxigenic strains, and longitudinally by month restricted to toxigenic strains only (**b**), with each ST indicated by color; STs are ordered from most common (bottom) to least common (top) and the proportion of samples from which toxigenic *C. difficile* was detected each month is depicted in the top line chart. For pairs of isolates of the same ST, the number of SNVs was calculated and evaluated in the context of epidemiological data to identify thresholds for identifying high-confidence transmission linkages. **c**, Genomic distance between isolates within patients (red) compared to between patients (blue); 95% of within-patient pairs were related within two SNVs. **d**, Genomic distance between isolates from patients with overlapping ICU admissions (red) versus none (blue); isolates from overlapping admissions were enriched at 0–1 SNVs. **e**, Genomic distance between isolates from patients ever hospitalized in the same single-patient room (red) versus never (blue); no association was observed between hospitalization in the same room and SNV distance.

**Extended Data Fig. 1:**
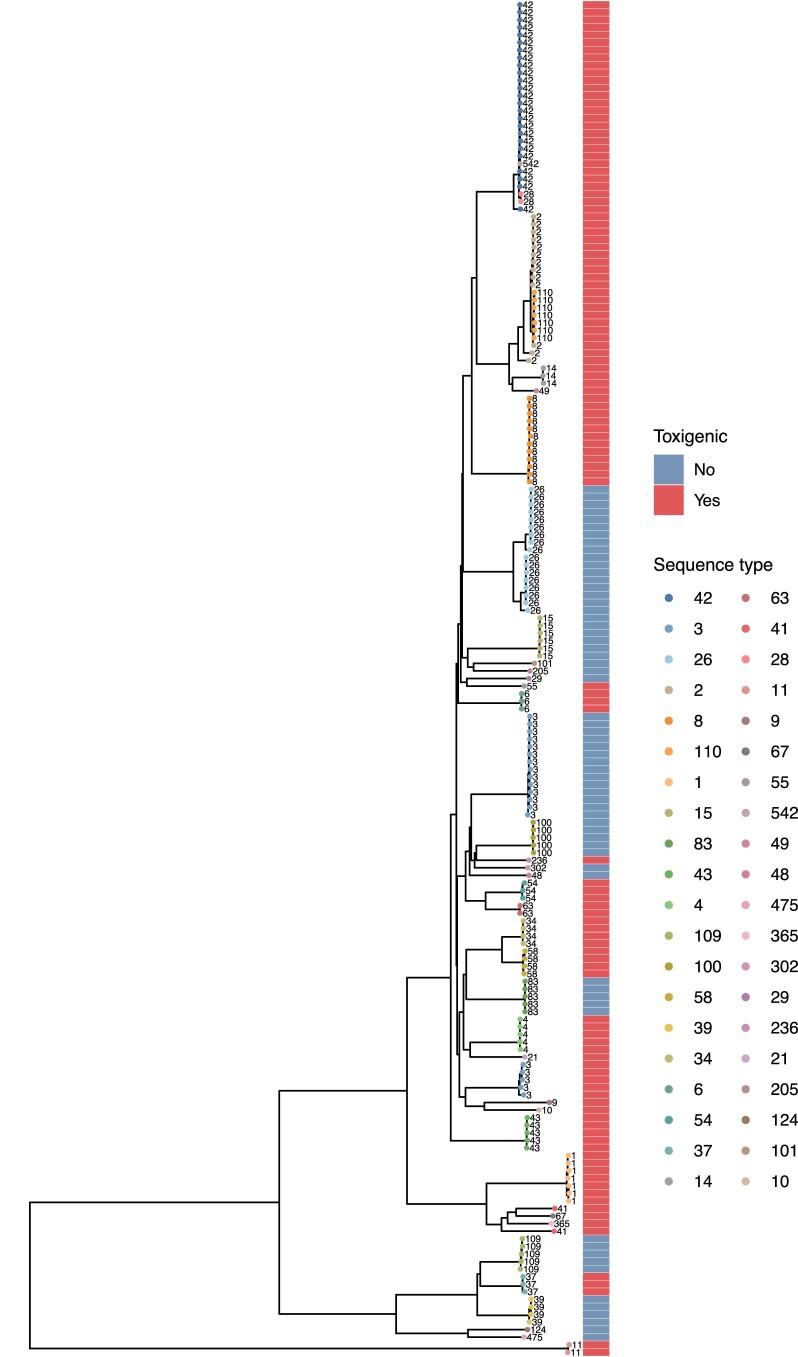
Whole-genome phylogeny of sequenced *C. difficile* isolates. Species-wide phylogenetic tree with Sequence Type (ST) and toxin status mapped onto the tips. Only the first isolate from a particular ST collected from a single patient is included.

**Extended Data Fig. 2:**
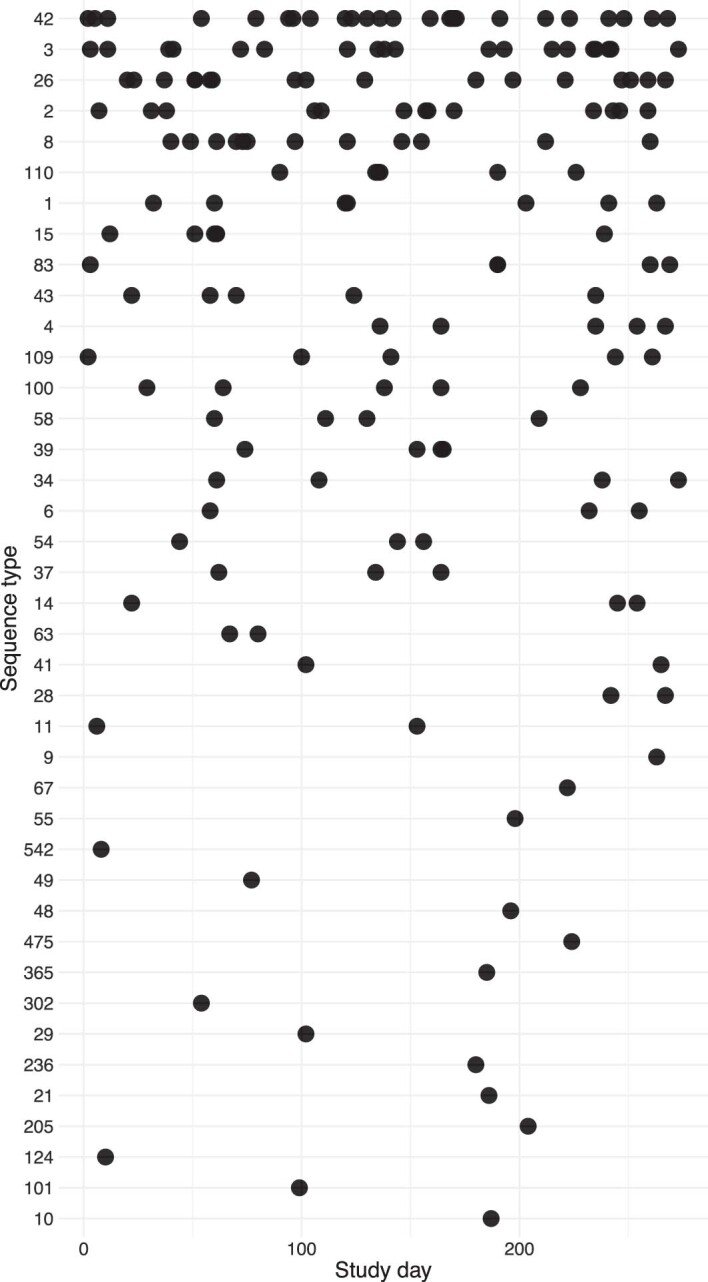
Sequence type identification over time. Distribution of sequence types of recovered *C. difficile* isolates over the study period, ordered from most common type (top) to least common type (bottom).

**Extended Data Fig. 3:**
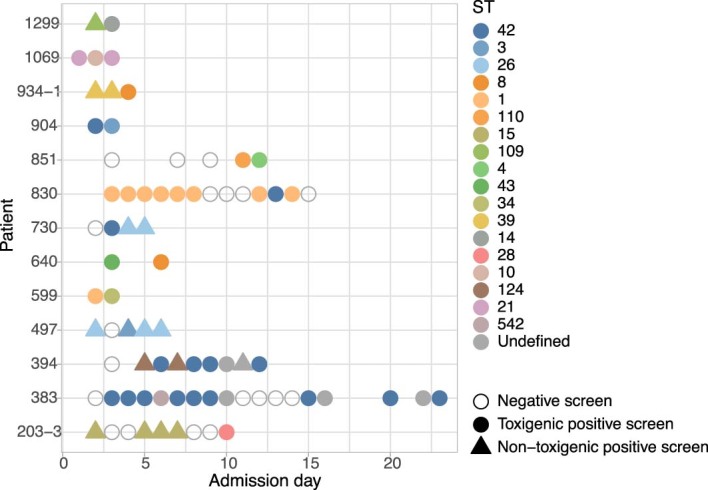
Culture pattern for patients with multiple sequence types detected over time. Dot plot depicting the 13 ICU admissions where more than one Sequence Type was recovered within a single admission.

We first tabulated and visualized the diversity of strains during the study period at sequence type (ST)-level resolution to evaluate evidence of dominant strains or temporal patterns consistent with within-hospital outbreaks or rapid spread of epidemic lineages. Among 179 unique patient–ST combinations, 40 unique STs were identified via in silico multi-locus ST (MLST). The most common ST identified was ST42 (14.0%) followed by ST3 (10.6%) and ST26 (9.5%). These STs are commonly associated with ribotypes 106 (ST42), 001 (ST3) and 015 (ST26.) A total of 116 (64.8%) strains had the *tcdA* or *tcdB* toxin loci detected in the WGS data and were defined as toxigenic (Fig. 2a). Toxigenic strains were distributed across the species-wide phylogenetic tree; only ST3 included both toxigenic and non-toxigenic strains (Fig. 2a and Extended Data Fig. 1). The composition of strain types was relatively stable over time; no emergence or disappearance of dominant STs was observed, a pattern that is more consistent with endemic spread of *C. difficile* as opposed to undetected outbreaks or epidemic lineages (Fig. 2b and Extended Data Fig. 2). Within patients, we observed that multiple strains were sometimes recovered from one admission; among 80 admissions with at least two *C. difficile* isolates and sequence-typed genomes recovered, 13 (15%) included multiple strain types: seven with at least two different toxigenic strains; five with a mix of toxigenic and non-toxigenic strains; and one with at least two different non-toxigenic strains (Extended Data Fig. 3). These additional strains could be secondarily acquired strains, either in addition to or after carriage of another strain, or they could be co-colonizing.

### Quantification of *C. difficile* importation and acquisition

**Extended Data Fig. 4:**
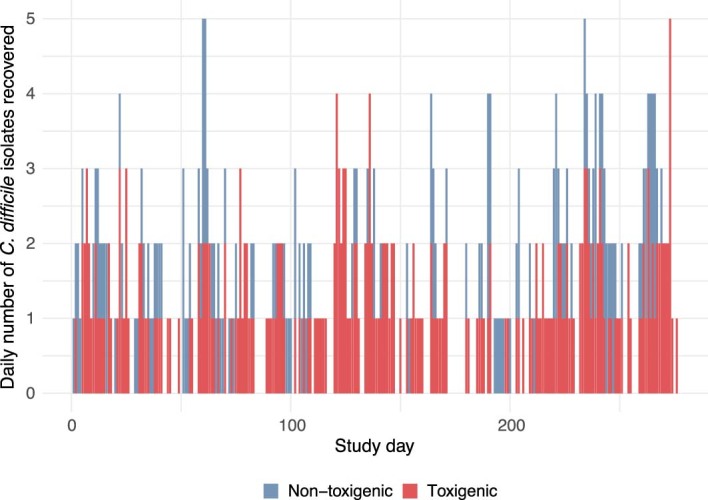
Daily *C. difficile* colonization burden during study period. Daily frequency of *C. difficile* detection in the ICU colored by toxigenic (red) vs. non-toxigenic (blue). Toxigenic *C. difficile* was recovered from at least one patient on 186/276 (67.4%) of sampled study days, while non-toxigenic *C. difficile* was recovered from at least one patient on 119/276 (43.1%) of sampled study days.

Next, we sought to characterize how common *C. difficile* carriage was in this setting by estimating the prevalence of toxigenic *C. difficile* importation and incidence of toxigenic *C. difficile* acquisition among ICU patients as inferred through enrichment culture and WGS to detect toxin genes. The overall screening prevalence (period prevalence) of toxigenic *C. difficile* during the whole study period was 9.3% (120/1,289 admissions). Among 1,141 admissions that qualified for importation analyses, 67 patients (5.9%) imported toxigenic *C. difficile*. Among 584 admissions who qualified for the acquisition analysis of toxigenic *C. difficile*, 32 patients cultured positive for an overall incidence rate of 1.6 acquisitions per 100 patient days. One acquisition was of the epidemic strain ST1, also known as ribotype 027. *C. difficile* was continuously detected in the unit during the study; a median of one patient hospitalized in the unit was identified as carrying toxigenic *C. difficile* on any given study day, and *C. difficile* was recovered from at least one patient in 186 (67.4%) out of 276 sampled study days (Extended Data Fig. 4). Finally, long-term carriage of toxigenic *C. difficile* strains was observed without development of *C. difficile* infection; three patients cultured positive for toxigenic *C. difficile* more than 8 weeks (56 days) apart—the standard cutoff for characterizing a *C. difficile* infection as new rather than recurrent. Of those patients, all three carried isolates of the same strain related within two single-nucleotide variants (SNVs) of the original strain.

Our cultivation of both toxigenic and non-toxigenic *C. difficile* from surveillance swabs enabled us to evaluate whether harboring a non-toxigenic strain was protective against subsequent acquisition of a toxigenic strain. Of the 584 admissions qualifying for acquisition analysis, there were four toxigenic *C. difficile* acquisitions among 27 non-toxigenic *C. difficile* importers (14.8%), compared to 28 toxigenic *C. difficile* acquisitions among 557 individuals with no *C. difficile* detected on admission (5.0%), with significantly more acquisitions among the non-toxigenic *C. difficile* carriers (chi-squared test *P* < 0.001). Thus, our data do not support non-toxigenic *C. difficile* as protecting against colonization with toxigenic strains; instead, they indicate that presence of non-toxigenic strains may be a marker of a gut environment conducive to toxigenic *C. difficile* acquisition. This interpretation is consistent with our detection of multiple *C. difficile* strains in 15% of longitudinally sampled patients with multiple sequenced isolates, as noted above.

### Genomic analysis of transmission in the ICU

While longitudinal culture-based screening uncovered presumed *C. difficile* acquisitions among patients hospitalized within the ICU, we next sought to understand how often asymptomatic *C. difficile* carriers were driving cross-transmission of *C. difficile* within the unit. To address this question, we investigated how many culture-based acquisitions were supported by genomic data, indicating cross-transmission from another colonized patient within the study. First, we defined a maximum genomic distance threshold consistent with recent transmission by examining two available data types: (1) within-patient genetic diversity from longitudinal samples; and (2) epidemiological data regarding plausible exposures between patients. We observed that a threshold of two SNVs would capture 95% of within-ST diversity observed in patients (Fig. 2c). To assess whether epidemiological data supported a two SNV threshold, we visually examined the SNV distance distributions among patients with overlapping ICU admissions and those who were ever in the same ICU room. We also calculated empirical *P* values by comparing true counts of genomic linkages among those with overlapping ICU admissions and same room exposure, to counts of linkages in randomly sampled datasets of the same size as the original dataset. Overlapping ICU hospitalizations were enriched among pairs of patients with isolates linked within two SNVs (Fig. 2d, empirical *P* = 0.001), while sequential occupation of the same hospital room was not statistically significantly associated with low SNV distances (Fig. 2e, empirical *P* value for two SNVs = 0.08). Together, these data, in addition to evidence from within-patient evolution from previous studies^13^, led us to choose a two SNV threshold as the initial criterion for defining the genomic linkages associated with plausible transmission.

**Fig. 3:**
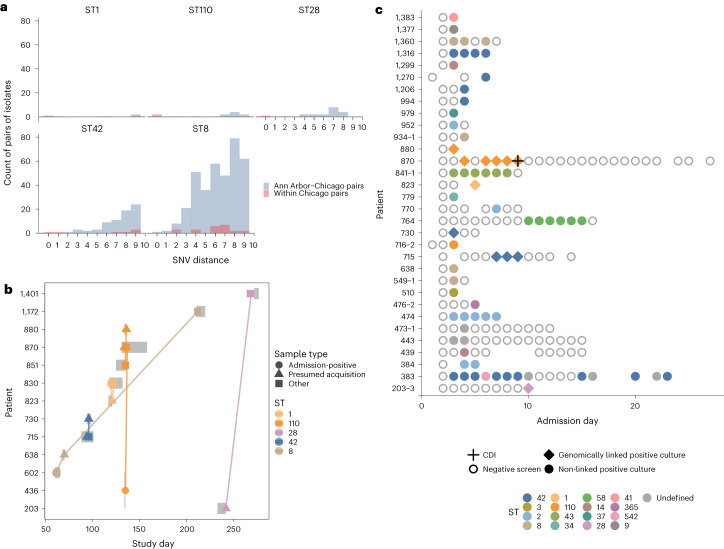
Interrogation of genomic linkages involving patients with culture-based acquisition of toxigenic *C. difficile*. **a**, Genetic distance between pairs of isolates from unique patients within the study (red) versus between study isolates and isolates from a geographically distinct collection (blue) among STs implicated in genetically linked acquisitions; ST8 shows patients commonly linked within two SNVs across geographical sites. **b**, Culture-based acquisitions of toxigenic *C. difficile* and their genomic linkages to other patient isolates. The gray boxes indicate ICU admission. The connecting lines indicate genomic linkages within two SNVs. ST8 isolates were linked across temporally distant hospitalizations. **c**, Culture-based acquisitions of toxigenic *C. difficile*. The empty circles indicate negative screens and the filled circles indicate positive screens; points are colored according to ST. Culture-based acquisitions genomically linked to another patient with high confidence are indicated by the diamonds; unlinked acquisitions are shown as circles. CDI, defined as a positive clinical PCR test for the toxin B gene, is indicated by a plus sign.

Applying a threshold of two SNVs to genomic linkages involving the 32 culture-based acquisitions of toxigenic *C. difficile* for which we had available sequences, seven (21.9%) were genomically linked to an isolate from another individual in the study. However, we further interrogated whether the two SNV threshold sufficiently supported cross-transmission by comparing these putatively acquired strains to isolates from an outside, contemporaneously collected but geographically distinct *C. difficile* isolate collection from Ann Arbor, MI (Methods). Four of the five STs included in genomically supported cross-transmission events were not linked across geographical sites at low SNV distances, bolstering our confidence that they could represent true within-ICU transmission events. However, one genomically linked ST, ST8, displayed close genetic relatedness across geographical sites, with isolates appearing just as likely to be linked within two SNVs across sites as within sites (Fig. 3a). Additionally, we noted that the ST8 strain was linked within two SNVs to a within-site strain collected 142 days later, further decreasing our confidence in the genomic support for cross-transmission of the ST8 strain (Fig. 3b). Thus, we determined that 6 (18.8%) of 32 culture-based acquisitions could be genomically linked with high confidence to another isolate within the unit (Fig. 3c). Of these six culture-based acquisitions with genomic linkages, only one acquirer was linked to a patient who was known to import *C. difficile* into the ICU, while two acquirers were genomically linked to each other and the other three acquirers were genomically linked to patients who did not qualify for importation analysis because they were not screened on the day of admission or the following day (Fig. 3b). Overall, while plausible transmission from *C. difficile* carriers was observed, the lack of robust genomic linkages involving culture-based acquirers suggests that cross-transmission among *C. difficile* carriers was not driving most observed *C. difficile* acquisitions in this study.

### Evidence of undetected *C. difficile* carriage on admission

**Fig. 4:**
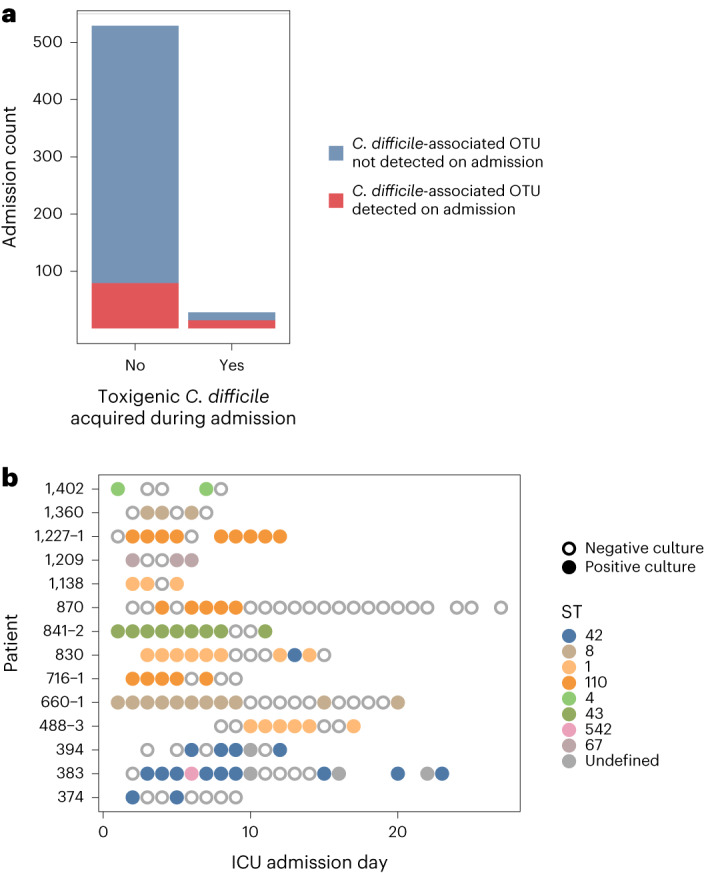
Potential undetected importations of *C. difficile* into the ICU. **a**, Detection of *C. difficile-*associated OTU from admission stool samples via 16S rRNA gene sequence analysis, stratified according to whether the patient subsequently acquired toxigenic *C. difficile* during their admission; *C. difficile-*associated OTUs were enriched among stool or rectal swab samples of patients who subsequently cultured positive for toxigenic *C. difficile* during their admission compared to those who did not (*P* = 4.5 × 10^−6^ via a two-sided chi-squared test). **b**, Dot plot depicting the 14 instances of intermittent toxigenic *C. difficile* detection among 38 admissions with at least three samples and with toxigenic *C. difficile* recovered from at least two samples.

The lack of concordance between culture-based acquisitions and genomically supported cross-transmission from *C. difficile* carriers within the ICU motivated exploration of potential explanations for this finding. Two main hypotheses underlying this discordance we considered were: (1) the unlinked acquisitions were largely acquired from unsampled patient or environmental reservoirs; or (2) the unlinked acquisitions were largely misclassified importations due to undetected carriage via false-negative admission screening cultures. With regard to the first hypothesis, we note that our genomic analyses would have detected reservoirs that caused more than one acquisition in this dataset via genomic linkage analyses, as was the case for two of six genomically linked acquisitions that were only linked to each other. Additionally, expanding the SNV threshold to five SNVs to account for more potential genomic diversity within undetected environmental or patient reservoirs would only lead to three more linkages among acquirers. However, multiple lines of evidence support the second hypothesis. First, in a 16S characterization of microbiome data from each admission screening stool sample, the best-match *C. difficile-*associated operational taxonomic unit (OTU) was more commonly detected in the negative admission screen of acquirers than in the negative admission screen among patients who did not subsequently culture positive for *C. difficile* (*P* < 0.001, Fig. 4a). Second, the daily sampling scheme revealed the common occurrence of intermittent detection of toxigenic strains; among 38 admissions with at least three samples and with toxigenic *C. difficile* recovered from at least two samples, 14 (36.8%) had at least one negative screen in between positive screens, supporting the plausibility of false-negative admission screens (Fig. 4b).

We hypothesized that patients with false-negative admission screens who had *C. difficile* detected by culture later in the ICU stay may have converted to culture positivity due to antibiotic-mediated disruption of the microbiota enhancing culture detection of *C. difficile*. To test this hypothesis, we focused on admission culture-negative patients who had the *C. difficile* OTU detected by 16S rRNA gene sequencing and evaluated whether those who had a subsequent specimen that cultured positive for toxigenic *C. difficile* were more likely to have received an antibiotic in the intervening time. All patients in this subset received one or more antibiotics during the periods of interest, so we focused our analysis on microbiota-disruptive and *C. difficile*-targeting antibiotics. While the presence of the *C. difficile*-associated OTU at the time of admission significantly increased the hazard ratio (HR) for subsequent detection of toxigenic *C. difficile* by culture, adding exposure to antibiotics most likely to disrupt the microbiota or antibiotics targeting *C. difficile* had no significant effect (Extended Data Table 1). One possible explanation for the lack of detectable role of antibiotics is the overall high rates of antibiotic use among patients in the unit (Table 1). Regardless of the potential causal role of antibiotic use in the putative unmasking of *C. difficile* colonization, these data are consistent with undetected importations, indicating that our culture-based assessments probably underestimate the importation prevalence and overestimate acquisition incidence of toxigenic *C. difficile*.

### Admission carriage of toxigenic *C. difficile* and CDI risk

**Fig. 5:**
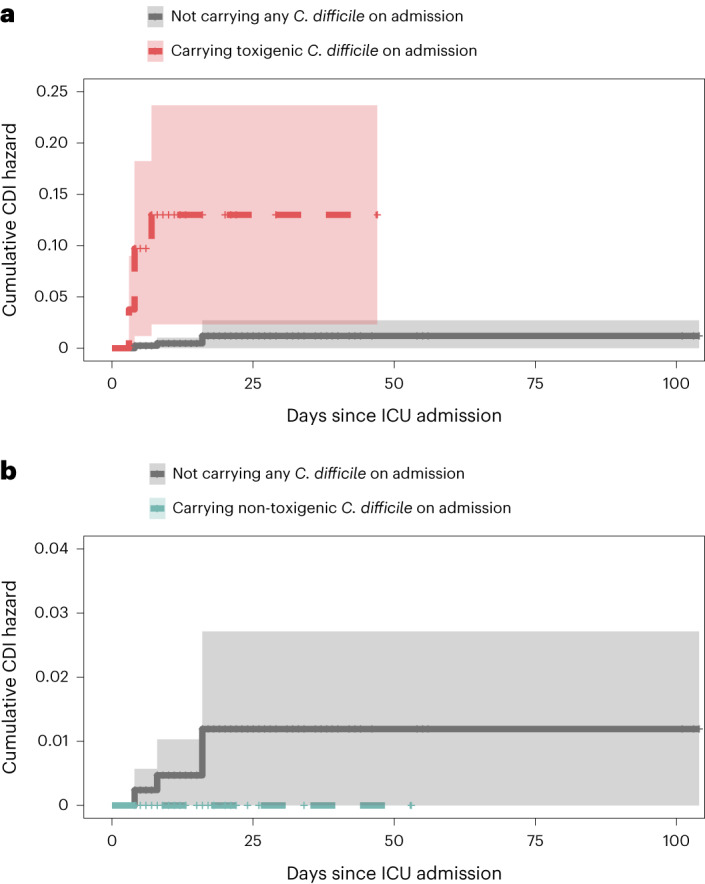
Risk of hospital-onset CDI based on admission colonization status. **a**,**b**, Cumulative hazard curves depicting the unadjusted risk of developing a healthcare-associated CDI during hospitalization among patients who carried toxigenic *C. difficile* on admission to the ICU (red) (**a**) compared to those who did not carry any (gray), and patients who carried non-toxigenic *C. difficile* on admission to the ICU (blue) compared to those who did not carry any (gray) (**b**). Patients carrying toxigenic strains on admission were at significantly increased risk of hospital-onset CDI compared to admission-negative patients (log-rank test, *P* = 3 × 10^−13^), while those carrying non-toxigenic strains showed no significant difference (log-rank test, *P* = 0.6). The thick lines indicate the estimated cumulative hazard in the designated group. The vertical lines indicate censored patients. The shaded areas indicate the 95% confidence bands.

While this study was focused on asymptomatic carriage and transmission of *C. difficile*, we also leveraged available data on clinical CDI to describe the relationships between *C. difficile* importation, *C. difficile* acquisition and healthcare facility-onset CDI as defined by the National Healthcare Safety Network (laboratory-identified CDI on day 4 or later of hospitalization)^14^. In total, we observed ten healthcare facility-onset CDI events after the day of ICU admission. Six of these events occurred among patients who imported toxigenic *C. difficile* into the ICU. Three events occurred in patients with culture-based toxigenic *C. difficile* acquisition, although only one of those putative acquisitions was genomically linked to another patient in the ICU (Fig. 3c). Finally, one event occurred in a patient from whom *C. difficile* was never recovered in a screening culture during their ICU stay. In an unadjusted survival analysis, patients who carried toxigenic *C. difficile* on admission to the ICU were significantly more likely to develop healthcare facility-onset CDI than patients who did not carry any *C. difficile* on admission (Fig. 5a; HR = 24.4, 95% confidence interval = 6.89–86.5, *P* < 0.001). Meanwhile, there was no statistically significant difference in the risk of developing healthcare facility-onset CDI among patients who carried non-toxigenic *C difficile* on admission compared to patients who did not carry any *C. difficile* on admission (Fig. 5b, *P* = 0.6); however, there were no healthcare facility-onset CDI events among patients carrying non-toxigenic *C. difficile* on admission, which precluded calculation of an HR and limited the robustness of this analysis. Together, these data are consistent with a stronger association between toxigenic *C. difficile* importation into the unit and healthcare facility-onset CDI than between within-ICU cross-transmission and healthcare facility-onset CDI.

## Discussion

Despite its notoriety as the leading cause of healthcare-associated infections in the United States^15^, there are major gaps in our understanding of the relative importance of different pathways leading to *C. difficile* infection in hospitals. In this study, we sought to improve our understanding of the contribution that patients asymptomatically carrying *C. difficile* on admission make to infections in a hospital unit. To this end we performed comprehensive admission and daily longitudinal culture screening for *C. difficile* carriage of virtually every patient admitted to a US-based ICU over a nine-month period. Our integrated genomic, microbiological and epidemiological analyses found that only 1% (6 of 584) of eligible patients admitted to the ICU during the study period had genomically supported acquisition of toxigenic *C. difficile* via cross-transmission. Together with the observed 24-times increased risk for developing CDI during hospitalization among patients colonized with toxigenic *C. difficile* on admission, these data suggest that interventions focused on preventing transition from colonization to overt infection will have a greater impact on further reducing the risk of CDI in this setting than investing additional resources aimed at interrupting cross-transmission.

While asymptomatic carriers of *C. difficile* can shed spores and transmit^9,16–20^, the relative importance of these patients as unmitigated reservoirs driving transmission—and the potential feasibility and utility of identifying carriers to prevent transmission—is unclear^12^. Our data show that although transmission of *C. difficile* leading to acquisition within the ICU probably occurred, it was uncommon, with only six genomically supported acquisitions over the nine-month study. Others argued that even though transmission from asymptomatic carriers may be rare when considered on a risk-per-carrier basis, the sizable minority of patients asymptomatically carrying *C. difficile* observed in this and other studies^8,11,17,19–26^ could still make screening for and isolating asymptomatic carriers an effective CDI prevention approach^22^. Indeed, one modeling evaluation and one quasi-experimental study supported the effectiveness of asymptomatic screening for reducing rates of CDI^27,28^. While the current study is not designed to directly address this question—and only includes screening on ICU admission rather than hospital admission—we note that among 67 asymptomatic importers of toxigenic *C. difficile* in this study only one was genomically linked to an acquisition that may have resulted in CDI during hospitalization in our nine-month study period. These observations suggest that implementation of basic infection prevention practices, such as hand hygiene and environmental cleaning and disinfection, in addition to this unit having only single-patient rooms, can minimize cross-transmission from asymptomatic carriers of *C. difficile* within the ICU. Notably, this observation might not hold in other molecular epidemiological contexts, such as when the epidemic hospital-associated strain ST1 (also known as ribotype 027 or NAP1) was observed to cause as many as 30% of *C. difficile* infections in the United States^29^. The application of routine molecular surveillance to monitor for the emergence of healthcare-associated epidemic lineages would allow for continual reassessment of the utility of admission screening for preventing within-unit transmission of *C. difficile*.

While our data do not support asymptomatic carriers posing a high risk to others, they support that the carriers themselves are at increased risk of CDI. In particular, our data are consistent with a growing body of evidence suggesting that carriers of *C. difficile* are more likely to develop CDI than noncarriers^11,25,30^. This is in contrast to the historically favored framework that carriage was protective for CDI^31^. Moreover, our data go further in also showing that carrying a non-toxigenic strain was associated with increased risk of acquiring a toxigenic strain, although a potentially protective role of non-toxigenic strains on the subsequent transition to infection could not be assessed due to limited power. While more studies are needed, some interventions that have shown promise in preventing CDI in carriers include administration of probiotics^32^, fidaxomicin prophylaxis^33^, fecal microbiota transplantation^34^ and antimicrobial stewardship programs^35^. Overall, the evidence that antibiotic prophylaxis and decolonization prevent CDI is mixed. Although antimicrobial stewardship is effective in reducing rates of CDI hospital-wide^36^, there is no specific evidence of its effectiveness among *C. difficile* carriers in particular^37^. It is also unclear whether targeting such interventions at *C. difficile* carriers would be more effective than horizontal application of antibiotic stewardship to all^38^. Others noted that identification of carriage might actually result in treatment that could further disrupt the microbiota^10^; our findings suggest that insensitivity of detection could be a limiting factor for successful application of targeted antibiotic stewardship interventions.

The results of this study should be interpreted in the context of certain limitations. First, the study setting was a single ICU in a single hospital in a high-income country, so we cannot generalize our findings to the entire hospital or to other healthcare settings, such as long-term care facilities, or to other countries. Furthermore, within this setting, ICU patients were housed in single rooms; cleaning with a sporicidal disinfectant was done daily and at the time of discharge unit-wide, both of which could reduce cross-transmission compared to other settings. Second, one colony was selected and sequenced per sample; thus, we likely missed co-colonizing strains^39–42^ that could have led to an underestimation of genomically supported transmission links. However, we leveraged daily longitudinal sampling to understand the diversity of strains recovered from a single admission; while multiple strains within an admission were observed, most admissions included only one strain type related within two SNVs. Third, our data suggest that imperfect sensitivity of *C. difficile* recovery could also have led to undetected *C. difficile* and thus some missed genomically supported transmission links. However, we also hypothesize that patients with detectable levels of *C. difficile* are more likely to transmit than patients with undetected levels of *C. difficile*. Fourth, we did not have isolates from clinically diagnosed CDI, so we could not verify whether colonizing strains were genomically matched to those from later infections, although the limited existing data from previous reports suggest high concordance between colonizing and subsequent infecting strain types^43^. Lastly, the hospital laboratory’s use of a *tcdB*-targeting PCR test alone for suspected CDI during the study could have resulted in overdiagnosis of CDI in patients who were colonized with toxigenic *C. difficile* and who had diarrhea from other causes. However, we note that several practices were in place during the study period to increase the positive predictive value of the PCR test, including rejection of formed stool specimens for PCR tests and implementation of provider alerts indicating that PCR tests should only be ordered when there are three consecutive unformed stools and the patient is not on a laxative.

In summary, applying admission and daily longitudinal screening for *C. difficile* within a US-based medical ICU, we found that while imported *C. difficile* strains were rarely transmitted to others, they were associated with significantly increased risk of infection in those who imported them. The low rate of transmission suggests that currently recommended infection prevention strategies are largely successful in preventing *C. difficile* cross-transmission from asymptomatic carriers in this setting. If replicated at other sites and in different types of healthcare settings, our findings have important implications for the prevention of CDI going forward. While current practices can limit cross-transmission, our results indicate that further reductions in hospital-onset CDI will require developing more effective strategies to interrupt the transition from colonization to clinical infection. Investigations to elucidate the origin and mechanism of acquisition and establishment of toxigenic *C. difficile* colonization outside the ICU setting are also warranted because the results may identify new targets to further reduce the burden of *C difficile* infection.

## Methods

### Patients and isolates

The study was reviewed and approved by the institutional review board at Rush University Medical Center with a requirement for verbal consent but waiver of written informed consent (Office of Research Administration no. 15122902). Posters describing the study and including the contact information for study investigators were posted in every patient room. Information sheets that included similar information were provided to the study subjects or their surrogates. The Institutional Review Board at the University of Michigan approved the study participants for the collection of contextual isolates used in transmission analysis (HUM00109057; see below). This single-center prospective observational study took place at Rush University Medical Center, a 676-bed tertiary care hospital in Chicago, Illinois. For a nine-month period between 3 April 2017 and 15 January 2018, all patients aged 18 years or older who were admitted to the 25-single-bed medical ICU had a rectal or stool swab collected on admission and every day during their stay until discharge from the ICU. Patients suspected by their treating physician to have CDI had a single stool sample tested by a commercial diagnostic PCR assay for *tcdB* (Xpert *C. difficile*, Cepheid); a positive result on this assay on day 4 or later of the hospital admission was considered a healthcare facility-onset CDI event in this study^14^. Patients with a positive test result were placed on contact isolation until at least 48 h after resolution of diarrhea. Several interventions were in place during the study to improve the positive predictive value of one-step PCR diagnostic testing for CDI on day 4 or later of the hospital stay: (1) between 11 January 2017 and 30 November 2017, a best practice alert appeared in the electronic medical record instructing healthcare providers who were placing an order for a PCR test to proceed only if the patient had three unformed stools in a 24-h period and was not receiving a laxative; (2) between 1 December 2017 and 11 January 2018, the best practice alert instructed providers to call the on-call infectious diseases specialist to obtain permission to order the test^44^. During the entire study period, the clinical laboratory rejected formed stool specimens for PCR testing. Environmental cleaning and disinfection was done daily and at the time of discharge in every ICU room using a sporicidal disinfectant. Clinical and demographic data for each enrolled patient were extracted from electronic medical records and bedside evaluation. Sex and gender were not considered in the study design, as all eligible admissions were included to enable comprehensive tracking of transmission and infection in the unit.

### *C. difficile* recovery and WGS

For the primary isolate collection from Rush University, samples of fresh stool (within 2 h of defecation) or rectal swab samples were collected using dual rayon swabs (BBL CultureSwab, Becton Dickinson) and transported in liquid Stuart medium on wet ice to the laboratory at Rush University Medical Center for processing. The first swab sample was placed in 1 ml tryptic soy broth containing 40% glycerol. A 150-μl aliquot was frozen at −80 and sent to the University of Michigan for culture of *C. difficile*. The second swab sample was frozen at −80° in the original container pending processing and nucleic acid extraction. Samples received by the University of Michigan were processed by a 24-h liquid culture enrichment in cefoxitin-cycloserine-fructose broth with sodium taurocholate (TCCFB) followed by plating any growth on cefoxitin-cycloserine-fructose agar with sodium taurocholate (TCCFA). Stool isolates of *C. difficile* were obtained at the University of Michigan via the following procedure. Stool specimens were screened for *C. difficile* by direct plating on TCCFA. If no growth was observed after 48 h, enrichment culture was attempted. This involved a 24-h liquid culture enrichment in TCCFB followed by plating any growth on TCCFA. See the Supplementary Methods and Supplementary Figs. 1 and 2 for details on validation of this culture enrichment protocol. Recovered *C. difficile* isolates underwent DNA extraction using the QIAGEN MagAttract Microbial DNA kit. Genomic libraries were prepared with the NEBNext Ultra DNA library prep kit and sequenced at the University of Michigan Advanced Genomics Core on an Illumina NovaSeq 6000, with 150-bp paired-end reads. All sequenced isolates have been deposited under BioProject nos. PRJNA821830 (Rush University collection) and PRJNA821832 (Michigan Medicine collection). Genomic libraries were prepared with the NEBNext Ultra DNA library prep kit and sequenced at the University of Michigan Advanced Genomics Core on an Illumina NovaSeq 6000 instrument.

An outside set of genomes generated from *C. difficile* infection isolates collected from patients in Ann Arbor, MI was also included for comparison. For this collection, between 11 February 2016 and 31 December 2017 stool specimens from patients diagnosed with CDI by the clinical microbiology laboratory at Michigan Medicine via a two-step algorithm that first detected *C. difficile* glutamate dehydrogenase and toxins A and B by enzyme immunoassay (EIA) (C. Diff Quik Check Complete, Alere) and reflexed to PCR for *tcdB* gene detection where the EIA results were discordant (BD GeneOhm assay from Becton Dickinson) were reserved for attempted *C. difficile* recovery and WGS.

### Genomic analyses

Raw sequencing reads were trimmed using Trimmomatic v.0.39 to remove adapters and low-quality bases^45^. Trimmed high-quality reads were then input into ARIBA v.2.14.6 (database downloaded in November 2021) to determine in silico MLST^46^. Sequencing reads were assembled using Spades v.3.15.3 and annotated using Prokka v.1.14.5 (refs. ^47,48^). The *tcdA* and *tcdB* genes were detected with BLAST v.2.11.0 using the complete *tcdA* and *tcdB* genes against the assembled contigs from the study collection. Assemblies were then used to make a species-wide core gene maximum-likelihood phylogenetic tree using the R package *cognac*^49^. From this phylogenetic tree, the previously characterized clades (clades 1–5 and cryptic) were clearly visible; each clade was identified by examining the characteristic strain types within each clade^50^. There were no isolates identified from clade 3. A single high-quality reference genome was selected from NCBI that corresponds to each of the clades (clade 1 = NZ_CP019870.1; clade 2 = NC_013316.1; clade 4 = FN668375.1; clade 5 = NC_017174.1); a clade 1 reference genome was used for isolates that fell into the cryptic clades. Trimmed sequencing reads were then mapped to each clade-specific reference genome using the Burrows–Wheeler Aligner-MEM v.0.7.17 and variants were called and filtered using Samtools v.1.11 (refs. ^51,52^). Only variants located at nucleotide positions present in all isolates in that clade (‘clade core’ variants) were considered for the transmission analyses. A threshold of two SNVs was used to identify putative in-unit cross-transmission, based on analysis of within-patient genetic diversity and comparison with epidemiological linkage (Results).

### Microbiome analyses

Rectal swab and stool samples from the Rush University collection also underwent DNA extraction and 16S rRNA analyses. The University of Michigan Microbiome Core extracted total DNA from feces using the MagAttract PowerMicrobiome kit (QIAGEN), and prepped DNA libraries as described previously^53^. Within each sequencing run samples were added randomly to the plates. Most 96-well plates included a positive control (ZymoBIOMICS Microbial Community DNA Standard, catalog no. D6306; Zymo Research) or a PCR-negative control. Because these samples did not have a low biomass, contamination with DNA isolation reagents was not a major concern and DNA isolation negative controls were not routinely included. DNA was amplified using dual-index primers targeting the V4 region of the 16S rRNA gene, as described previously^54^. Sequencing was conducted on the Illumina MiSeq platform using the MiSeq Reagent kit v3 for a total of 500 total cycles, with modifications found in the Schloss standard operating procedure (https://github.com/SchlossLab/MiSeq_WetLab_SOP). The quality control steps in our 16S rRNA gene sequencing data processing protocol using mothur v.1.43.0 included: (1) removal of sequences with ambiguous bases after alignment of paired sequences; (2) removal of sequences longer than 275 nucleotides; and (3) removal of chimeras. After processing, samples with fewer than 3,000 sequence reads were excluded from the analysis. The 16S rRNA gene sequence data were processed using mothur^54,55^. After sequence processing and alignment to the SILVA reference alignment (release 132)^56^, sequences were binned into OTUs based on 97% sequence similarity using the OptiClust method^57^. To identify the OTU corresponding to *C. difficile*, the 16S ribosomal RNA gene from the strain VsPI 10463 partial sequence (GenBank accession no. AF072473) was aligned with representative sequences for the 500 most abundant OTUs in the full MAriMba dataset using standard nucleotide BLAST (blastn). The top hit was a 99% match to the representative sequence from OTU00089.

### Statistical analyses

Enrichment of overlapping ICU admissions and sequential stays in the same room at different SNV distances were compared by generating 1,000 random datasets with the same size as the original dataset, with replacement and calculating empirical *P* values using the formula (*r* + 1)/(*n* + 1) where *r* is the number of replicates that produce a test statistic greater than or equal to the one calculated from the real data and *n* is the number of replicates. The proportion of *C. difficile-*associated OTUs among negative admission screens versus presumed acquirers were compared using a chi-squared test. A Cox proportional hazards model with right censoring was used to calculate unadjusted HRs comparing the time from ICU admission to a healthcare-associated CDI between admissions in which importation of *C. difficile* was detected and admissions in which *C. difficile* was not detected. Cox proportional hazards models were also used to assess the association of antibiotic exposure during the ICU stay and risk of acquisition of toxigenic *C. difficile* using culture. Antibiotic exposures were stratified a priori into three groups: any antibiotic; antibiotic deemed high-risk for *C. difficile* infection^58^; and antibiotic with clinical activity against *C. difficile* (oral vancomycin, metronidazole, fidaxomicin). Separate models included antibiotic exposure as a dichotomous or time-varying covariate. An interaction between antibiotic exposure and presence of the *C. difficile* OTU at the time of ICU admission was also assessed. All analyses were performed in R v.4.0.2 or SAS v.9.4.

### Reporting summary

Further information on research design is available in the Nature Portfolio Reporting Summary linked to this article.

## Online content

Any methods, additional references, Nature Portfolio reporting summaries, source data, extended data, supplementary information, acknowledgements, peer review information; details of author contributions and competing interests; and statements of data and code availability are available at 10.1038/s41591-023-02549-4.

### Supplementary information

Supplementary Information Supplementary Figs. 1 and 2 and Methods.

Reporting Summary

## Figures and Tables

**Extended Data Table 1:**
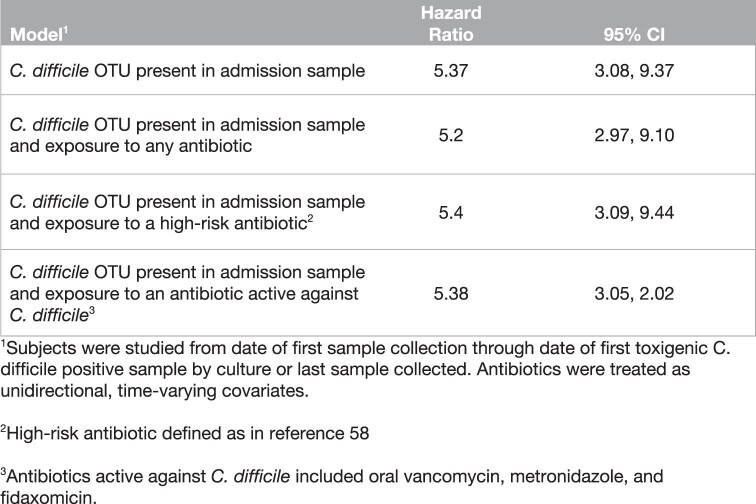
Association of antibiotic exposure and acquisition of toxigenic *C. difficile* by culture during ICU stay in individuals who carried the *C. difficile* OTU at the time of ICU admission

## Data Availability

The raw *C. difficile* genome sequencing data, along with patient IDs, has been deposited in the NCBI under BioProjects nos. PRJNA821830 and PRJNA821832. The 16S rRNA gene sequence data, along with patient IDs and the order of the longitudinal samples has been submitted to the NCBI under BioProject no. PRJNA875659. A single high-quality reference genome was selected from the NCBI for variant calling that corresponds to each of the clades (clade 1, NZ_CP019870.1; clade 2, NC_013316.1; clade 4, FN668375.1; clade 5, NC_017174.1).

## Code availability

The custom analysis pipelines for genomic variant calling and filtering are available on GitHub (https://github.com/alipirani88/snpkit).
