# Supplementary material for: Longitudinal genomic surveillance of carriage and transmission of Clostridioides difficile in an intensive care unit
Source: Nat Med. 2023 Sep 18;29(10):2526–34. doi: 10.1038/s41591-023-02549-4 (PMC10579090; doi:10.1038/s41591-023-02549-4)

nature medicine

Article

https://doi.org/10.1038/s41591-023-02549-4

Longitudinal genomic surveillance of carriage and transmission of *Clostridioides difficile* in an intensive care unit

In the format provided by the authors and unedited

Supplementary Information

Evaluation of sensitivity of spore cultivation from stool (Figure S1)

Comparison of relative sensitivity of toxigenic culture and PCR for detection of C. difficile
Generation of spore stocks. Spore stocks were prepared for one lab strain (630, ATCC BAA-1382) and two clinical isolates (one of ribotype 027 and one 014-020) of *C. difficile*. Each strain was plated onto taurocholate-cycloserine-cefoxitin-fructose agar (TCCFA) and incubated for 24 ±6 hours at 37°C in an anaerobic chamber. All incubations were performed at this temperature and for this period of time unless otherwise stated. An inoculating needle was used to inoculate single colonies into each of four vials containing 5 mL Columbia broth and then incubated again. The entire contents of these vials was combined with 40 mL Clospore media and incubated again. At five days of growth, cultures were checked daily for sufficient sporulation with phase-contrast microscopy.

After ten days, Clospore tubes were centrifuged at 2393g for 20 min at 4°C. The resulting spore pellet was resuspended in 40 mL cold sterile water and centrifuged again. This washing step was repeated once with 0.05% Tween 20 and an additional three times with sterile water only. The final pellets were suspended in 1.0 mL sterile water, transferred to polypropylene cryogenic vials, and stored at 4°C.

Quantification of spore stocks. To determine the concentration of spores in each prepared stock, 10 µL spore stock was pipetted into a cryogenic tube and 990 µL Ultra Pure Distilled Water was added. After vortexing, the vial was heat shocked at 65°C for 20 minutes on a heating block to eliminate vegetative cells and non-viable spores. In separate cryogenic tubes, the spores were serially diluted by a factor of 10^{-5} using pre-reduced PBS as the diluent. Under anaerobic conditions 100 µL of each dilution was plated in triplicate on TCCFA and incubated. Colonies were visually counted and, if necessary, dilution factors were adjusted to ensure CFUs per plate were between 30 and 300.

Preparation of C. difficile spores in stool. In cryogenic tubes, PBS was used to serially dilute known concentrations of spore stock by a factor of 10^{-8}. In separate cryogenic tubes, approximately 0.5 g stool was aliquoted and mixed with various concentrations of the diluted spore stocks and PBS to final volumes of 1 mL and spore concentrations from 100 to 0.001 CFU/µL. Stool specimens for creating the standards were selected based on the criteria that they tested negative in the clinical microbiology laboratory for *C. difficile* by a Simplexa™ RT-PCR for *tcdB* (Focus Diagnostics, Cypress, California) and by anaerobic culture (on TCCFA or in TCCFB) after an ethanol shock. The resulting stool standards were mixed and stored at overnight at 4°C, prior to analysis by anaerobic enrichment culture and RT-PCR.

Determining the limit of detection by anaerobic enrichment culture. In an anaerobic chamber, the spore preparations in stool were mixed with a 10-µL inoculating loop that was used to incubate 5 mL TCCFB before incubation for 24–48 hours (checked at 24 hours, and reincubated if no growth on visual inspection). The broth enrichment cultures were vortexed, diluted in pre-reduced PBS and then plated in triplicate onto TCCFA, and incubated. The plates were then viewed for *C. difficile* growth, assigning the limit of detection at the stool standard concentration that *C. difficile* no longer grew.

Determining limit of detection for C. difficile by RT-PCR. The spore preparation in stool were tested in the Michigan Medicine clinical microbiology lab. The lab utilizes the Simplexa™ RT-PCR for *tcdB* (Focus Diagnostics, Cypress, California) assay for molecular detection of *C. difficile*. The limit of detection was assigned at the stool standard concentration immediately before *C. difficile* was not detected by this RT-PCR assay.

Limit of detection by broth enrichment and RT-PCR. The limits of detection via broth enrichment culture for strains 630, R027, and R014-020 were 0.001, 0.001, and 0.01 CFU/µL, respectively (**Figure S2**). Testing the same spore preparations in stool, the limits of detection using the RT-PCR for tcdB for strains 630, R027, and R014-020, RT-PCR limits of detection were 1, 10, and 1 CFU/µL, respectively (**Figure S2**). All positive, negative, and internal controls indicated that the procedure was carried out and did not suggest the presence of any contamination.

Figure S1 - Experimental process for evaluation of protocol for *C. diffcile* cultivation from stool. Broth cultures were incubated overnight before plating. RT-PCR for tcdB was performed as part of the diagnostic process for C. difficile at Michigan Medicine's clinical laboratories; stool standards for each strain were prepared as usual before being delivered for testing.

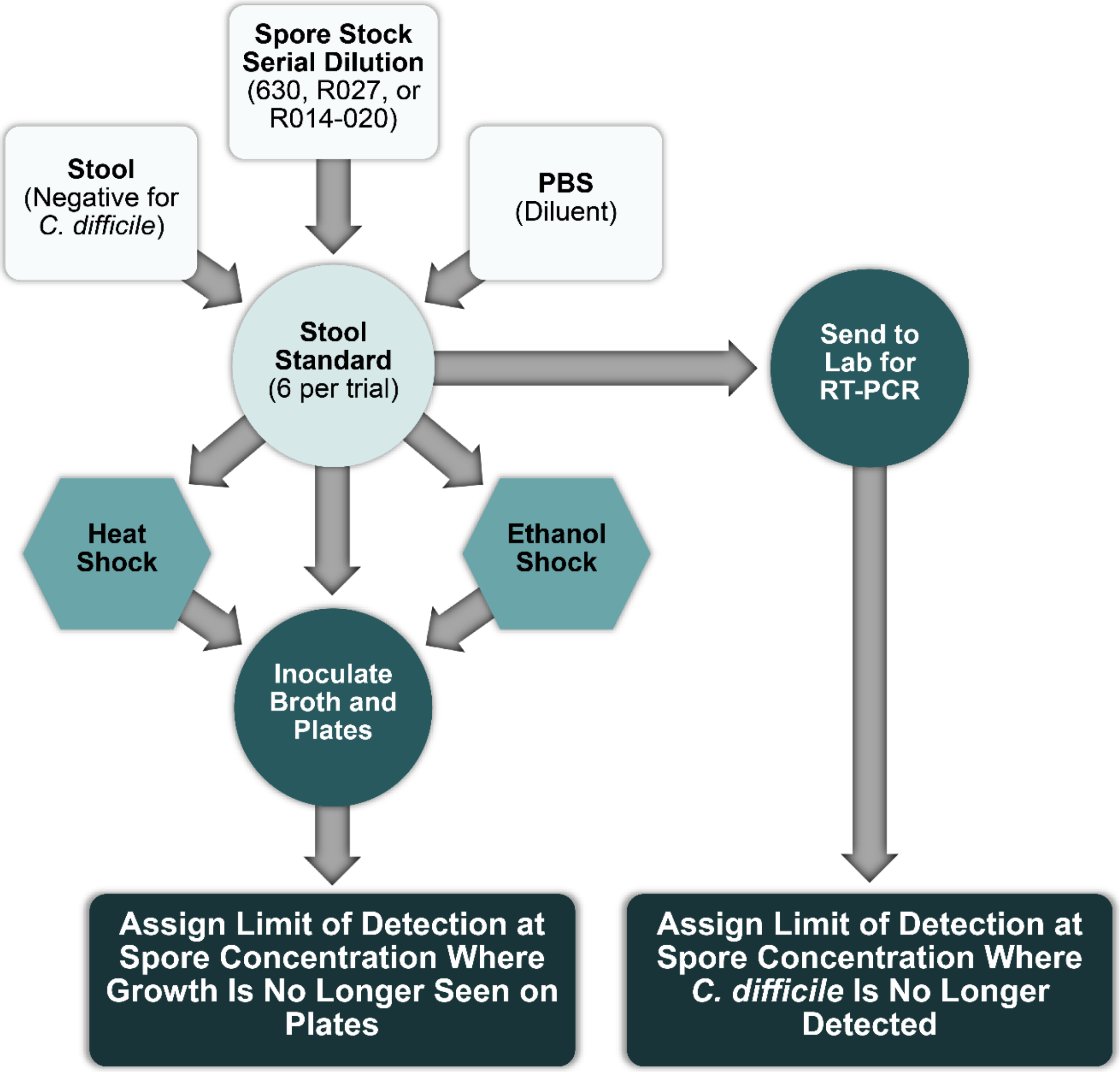

Figure S2. (A) Anaerobic culture LODs by direct plating. (B) Anaerobic culture LODs with a broth enrichment step. (C) RT-PCR for *tcdB* LODs. All charts show limits of detection in CFU/μL on the vertical axis (logarithmic scale). Each bar represents results from one triplicate trial.

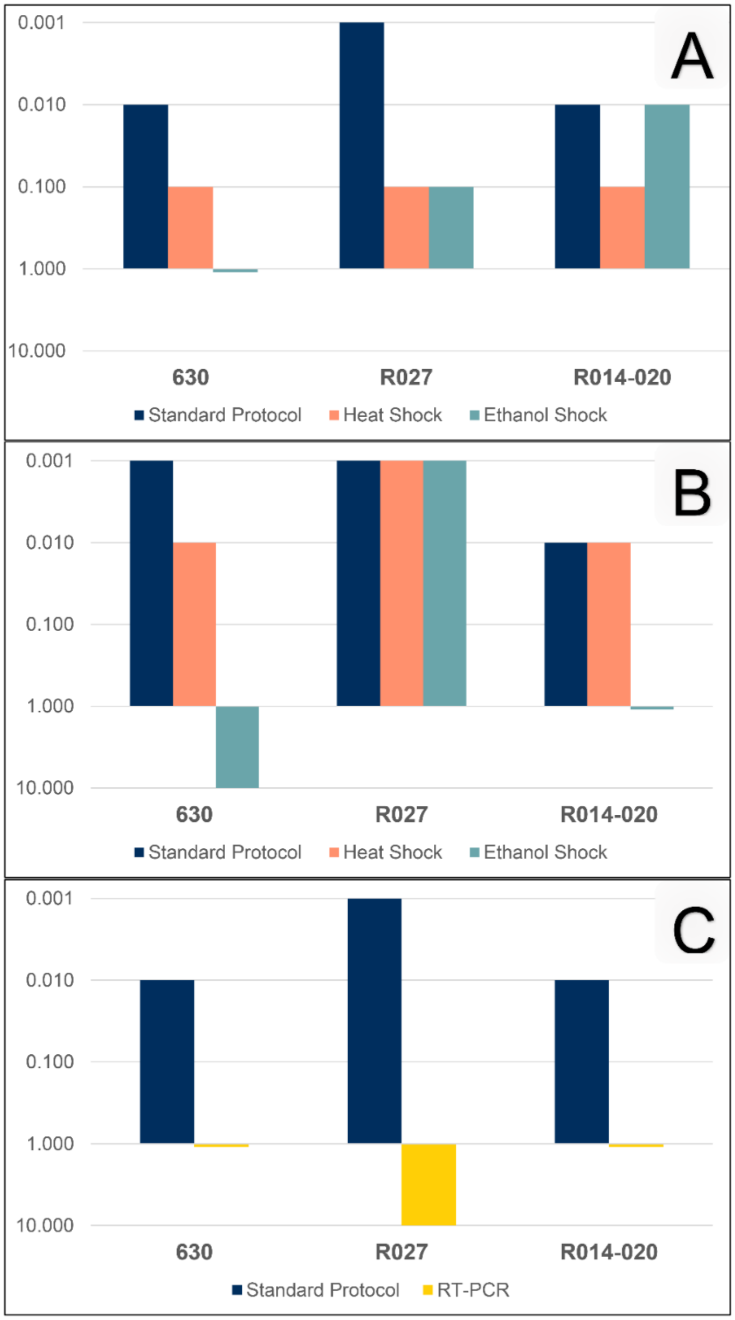

Supplement: Supplementary file 1 — Supplementary Figs. 1 and 2 and Methods. [file 41591_2023_2549_MOESM1_ESM.pdf]
