# Supplementary material for: Longitudinal genomic surveillance of carriage and transmission of Clostridioides difficile in an intensive care unit
Source: Nat Med. 2023 Sep 18;29(10):2526–34. doi: 10.1038/s41591-023-02549-4 (PMC10579090; doi:10.1038/s41591-023-02549-4)
Supplement: Supplementary file 2 — Reporting Summary [file 41591_2023_2549_MOESM2_ESM.pdf]

nature portfolio

Corresponding author(s): Evan Snitkin

Last updated by author(s): 8-14-2023

Reporting Summary

Nature Portfolio wishes to improve the reproducibility of the work that we publish. This form provides structure for consistency and transparency in reporting. For further information on Nature Portfolio policies, see our Editorial Policies and the Editorial Policy Checklist.

Statistics

For all statistical analyses, confirm that the following items are present in the figure legend, table legend, main text, or Methods section.

n/a	Confirmed	
☐	☒	The exact sample size (*n*) for each experimental group/condition, given as a discrete number and unit of measurement
☐	☒	A statement on whether measurements were taken from distinct samples or whether the same sample was measured repeatedly
☐	☒	The statistical test(s) used AND whether they are one- or two-sided<br>*Only common tests should be described solely by name; describe more complex techniques in the Methods section.*
☒	☐	A description of all covariates tested
☐	☒	A description of any assumptions or corrections, such as tests of normality and adjustment for multiple comparisons
☐	☒	A full description of the statistical parameters including central tendency (e.g. means) or other basic estimates (e.g. regression coefficient) AND variation (e.g. standard deviation) or associated estimates of uncertainty (e.g. confidence intervals)
☐	☒	For null hypothesis testing, the test statistic (e.g. *F*, *t*, *r*) with confidence intervals, effect sizes, degrees of freedom and *P* value noted<br>*Give P values as exact values whenever suitable.*
☒	☐	For Bayesian analysis, information on the choice of priors and Markov chain Monte Carlo settings
☒	☐	For hierarchical and complex designs, identification of the appropriate level for tests and full reporting of outcomes
☒	☐	Estimates of effect sizes (e.g. Cohen's *d*, Pearson's *r*), indicating how they were calculated

Our web collection on statistics for biologists contains articles on many of the points above.

Software and code

Policy information about availability of computer code

Data collection	No software was used.
Data analysis	Open source genomic analysis software included: 1) trimmomatic 0.39, 2) ARIBA 2.14.6, 3) BLAST 2.11.0, 4) cognac (from github 10/18/2021), 5) bwa-mem 0.7.17, 6) samtools 1.11, 7) Spades 3.15.3, 8) prokka 1.14.5 and 9) R v4.0.2.<br><br>16S analysis was conducted with mothur v.1.43.0, 2.<br><br>Code was written in R to: 1) generate figures and 2) perform statistical analyses.

For manuscripts utilizing custom algorithms or software that are central to the research but not yet described in published literature, software must be made available to editors and reviewers. We strongly encourage code deposition in a community repository (e.g. GitHub). See the Nature Portfolio guidelines for submitting code & software for further information.

Data

Policy information about availability of data

All manuscripts must include a data availability statement. This statement should provide the following information, where applicable:

- Accession codes, unique identifiers, or web links for publicly available datasets
- A description of any restrictions on data availability
- For clinical datasets or third party data, please ensure that the statement adheres to our policy

The raw C. difficile genome sequencing data, along with patient ID has been deposited in NCBI under BioProjects PRJNA821830 and PRJNA821832. 16S rRNA gene sequence data, along with patient ID and the order of longitudinal samples has been submitted to NCBI under bioproject PRJNA875659. A single high-quality reference genome was selected from ncbi for variant calling that corresponds to each of the clades (clade 1 = NZ_CP019870.1, clade 2 = NC_013316.1, clade 4 = FN668375.1, clade 5 = NC_017174.1).

Human research participants

Policy information about studies involving human research participants and Sex and Gender in Research.

Reporting on sex and gender	Sex was not considered for enrollment, and is reported in aggregate here based on extraction from electronic medical records and bedside evaluation.
Population characteristics	This study took place at Rush University Medical Center, a 676-bed tertiary-care hospital in Chicago, Illinois. For a 9-month period between April 3, 2017 and January 15, 2018, all patients age 18 years or older that were admitted to the 25-bed medical intensive care unit (ICU) had a rectal or stool swab collected upon admission and every day during their stay until discharge from the ICU.
Recruitment	All individuals with with one or more surveillance swabs were included in this study. 375/1,516 admitted patients were excluded either due to declining swab, or relevant clinical criteria (see Figure 1 for patient flow diagram). There is no bias in exclusion that we anticipate impacts the conclusions of this study.
Ethics oversight	The study was reviewed and approved by the Institutional Review Board at Rush University Medical Center with a requirement for verbal consent but waiver of written documentation of informed consent (ORA# is 15122902). Posters describing the study and including contact information for study investigators were posted in every patient room. Information sheets that included similar information were provided to study subjects or their surrogates. The Institutional Review Board at the University of Michigan approved the study protocol for the collection of contextual isolates used in transmission analysis (HUM00109057).

Note that full information on the approval of the study protocol must also be provided in the manuscript.

Field-specific reporting

Please select the one below that is the best fit for your research. If you are not sure, read the appropriate sections before making your selection.

☐ Life sciences ☐ Behavioural & social sciences ☒ Ecological, evolutionary & environmental sciences

For a reference copy of the document with all sections, see nature.com/documents/nr-reporting-summary-flat.pdf

Ecological, evolutionary & environmental sciences study design

All studies must disclose on these points even when the disclosure is negative.

Study description	Prospective observational study of C. difficile carriage in a medical intensive care unit.
Research sample	This study took place at Rush University Medical Center, a 676-bed tertiary-care hospital in Chicago, Illinois. For a 9-month period between April 3, 2017 and January 15, 2018, all patients age 18 years or older that were admitted to the 25-bed medical intensive care unit (ICU) had a rectal or stool swab collected upon admission and every day during their stay until discharge from the ICU.
Sampling strategy	Daily rectal or stool swabs for all patients in medical ICU.
Data collection	Clinical data was collected by study authors at bedside or through query of electronic health records.
Timing and spatial scale	Daily samples between April 3, 2017 and January 15, 2018.
Data exclusions	•Inclusion criteria:<br>–Adult MICU patient (≥ 18 yo)<br>–≥2 rectal swabs or fecal samples collected<br>•Exclusion criteria:

–Patients with a colostomy or ileostomy
–Patients with a fecal management device
–Patients with a history of inflammatory bowel disease
–Patients with a known laboratory diagnosis of an acute viral or bacterial gastroenteritis or infectious diarrhea, including CDI, in the 2 weeks prior to MICU admission.
–Patient with history of fecal microbiota transplantation
–Patient with active GI bleeding
--Patient who is neutropenic and who cannot provide a fecal sample (rectal swabs weren't done on neutropenic patients)
-- Patient clinically unstable, aggressive, or combative, receiving comfort care (dying), undergoing a procedure, out of room on multiple visits
-- Patient declined study participation

Reproducibility	Data integrity was validated at multiple steps, including: 1) inclusion of positive and negative controls in 96-well sequencing plates, 2) QC of sequence to verify that no contamination or between-well transfer occurred and 3) verifying that the overall increased genetic similarity of C difficile within patients versus between patients was observed.
Randomization	This was a descriptive observational study, without allocations to groups.
Blinding	Samples and data were collected by investigators at Rush University medical center, with IDs assigned prior to data sharing, or subsequent analysis at the University of Michigan.

Did the study involve field work? ☐ Yes ☒ No

Reporting for specific materials, systems and methods

We require information from authors about some types of materials, experimental systems and methods used in many studies. Here, indicate whether each material, system or method listed is relevant to your study. If you are not sure if a list item applies to your research, read the appropriate section before selecting a response.

Materials & experimental systems

n/a	Involved in the study	
☒	☐	Antibodies
☒	☐	Eukaryotic cell lines
☒	☐	Palaeontology and archaeology
☒	☐	Animals and other organisms
☐	☒	Clinical data
☒	☐	Dual use research of concern

Methods

n/a	Involved in the study	
☒	☐	ChIP-seq
☒	☐	Flow cytometry
☒	☐	MRI-based neuroimaging

Clinical data

Policy information about clinical studies
All manuscripts should comply with the ICMJE guidelines for publication of clinical research and a completed CONSORT checklist must be included with all submissions.

Clinical trial registration	N/A
Study protocol	The parent study during which samples were collected is in preparation for submission and is summarized in the methods section. Additional details can be provided upon request.
Data collection	This study took place at Rush University Medical Center, a 676-bed tertiary-care hospital in Chicago, Illinois. For a 9-month period between April 3, 2017 and January 15, 2018, all patients age 18 years or older that were admitted to the 25-bed medical intensive care unit (ICU) had a rectal or stool swab collected upon admission and every day during their stay until discharge from the ICU.
Outcomes	Outcomes here were C. difficile culture positivity for carriage and commercial PCR diagnostic for infection.
